# Editorial: Vaccine education and promotion

**DOI:** 10.3389/fpubh.2025.1610968

**Published:** 2025-04-29

**Authors:** Graça S. Carvalho, Carlos Alberto de Oliveira Magalhães Júnior

**Affiliations:** ^1^Research Centre for Child Studies, University of Minho, Braga, Portugal; ^2^State University of Maringá, Maringá, PR, Brazil

**Keywords:** determinants of vaccination, vaccination hesitancy, vaccination among students, health education, health promotion

This editorial highlights key developments in the Public Health Research Topic “*Vaccine education and promotion*.” This critical area aims to inform and encourage vaccination by providing accurate scientific information and implementing effective vaccination programs, emphasizing their benefits, safety, and role in preventing infectious diseases. A total of 39 manuscripts were submitted for consideration. Following a rigorous peer-review process and revisions based on expert feedback, 29 (74.3%) were accepted for publication. Among these, 14 explore the determinants of vaccination, eight examine vaccine hesitancy and seven address vaccination among students.

## 1 Determinants of vaccination

The determinants of vaccination are diverse but can be broadly categorized into three groups (1): (i) Contextual factors, including historical, socio-cultural, environmental, health system/institutional, economic, and political influences; (ii) Individual and group influences, such as perceptions of vaccines and the impact of social or peer environments; (iii) Vaccine- and vaccination-specific Research Topic, factors directly related to the vaccine or the act of vaccination itself. Vaccination uptake also varies across risk populations, with distinct determinants potentially influencing behavior in each group (2). As such, a thorough understanding of these factors is essential for designing targeted interventions to improve immunization coverage (3). The 13 papers included in this section, *Determinants of Vaccination*, are organized across three Research Topic:

### 1.1 Seven papers look at communication and engagement for vaccination

Chang et al. compared USA public vaccination decisions for newly-developed and established vaccines, and recognized the need for clear communication and community engagement as critical strategies for addressing public concerns and misinformation; Rahman et al. looked at the perceptions of Nigerian persons with disabilities regarding the COVID-19 pandemic and the vaccine and identified the need for culturally and religiously sensitive communication strategies, and tailored educational programs by social workers; Xu et al. analyzed “vaccine science popularization” in the Chinese social media Weibo during the COVID-19 pandemic where publishers were divided into individuals, organizations, media, government, and scientists, and verified that Weibo scientists' arguments were those that more positively influenced the effect of vaccine popularization; Hijazi et al. identified the Israeli Ministry of Health communication strategies regarding vaccines during COVID-19 pandemic and how healthcare workers shaped their professional socialization processes within the health system, leading to a reliance on established communication strategies and informational channels; Shumba et al. analyzed community health volunteers experiences of implementing COVID-19 *Vaccine education and promotion* in Kenya during the pandemic, showing they contributed to the high uptake of primary vaccines and boosters; Wrenger et al. used a protocol (INFORMed) in Germany to identify the wish for advice in hesitant and no-hesitant new-born's parents and the comparison of parents in terms of their respective information needs; and Smith et al. addressed a community engagement framework that can provide a roadmap to navigate the dynamic and multifaceted nature of equity-related work by paving the way for meaningful interventions to mitigate health disparities.

### 1.2 Four papers look at socioeconomics discrepancy

Tao et al. found substantial racial and socioeconomic disparities in influenza vaccination uptake among United States adults aged 65 years or older; Zhao et al. investigated the extent of influenza vaccine coverage in south China adults aged 60 years or older and identified the factors influencing vaccine uptake; Ramphul et al. identified areas in Texas, USA, with high and low HPV vaccination rates and explored differences in neighborhood characteristics, showing that vaccination coverage rates depend on the community's income level; and Ye and Ting Su explored the factors related to public health campaigns, in Wuxi region of China, that can improve vaccination rates in low socioeconomic groups and rural areas, to contributing to better public health strategies.

### 1.3 Three papers look at children's immunization

Alami et al. analyzed the Canadian survey about parents/guardians' perspectives on influenza immunization and identified the main factors influencing low rates of children's vaccination, such as residing in rural areas, lower parental education and lower household income; Dires et al. looked at the factors influencing childhood immunization status in East Africa, which varied among countries and regions, and found that mothers attending antenatal care played a key role in children's vaccination; and Assefa et al. evaluated the determinants of pneumonia conjugate vaccine (PCV) dropout among children aged 12–23 months in Ethiopia and identified the significant factors influencing PCV dropout, such as having a health card, having received the PCV 2 vaccinations, and region.

## 2 Vaccination hesitancy

Vaccine hesitancy refers to the delay in acceptance or outright refusal of vaccines despite the availability of vaccination services; it is a complex and context-specific phenomenon that varies across time, location, and type of vaccine (4). It has been linked to declining vaccination coverage and a heightened risk of outbreaks and epidemics of vaccine-preventable diseases (5). The eight papers in the *Vaccination Hesitancy* section are distributed across two Research Topic:

### 2.1 Five papers address vaccination hesitancy in the community

Osaghae et al. leveraged long-standing community-academic partnerships in two cities to develop a curriculum for interventions to decrease COVID-19 vaccine hesitancy within Black communities in the USA; Muis et al. examined the effectiveness of three different messages for persuading Canadian individuals to get vaccinated against COVID-19, and the role that emotions play in persuasion, verifying that emotions mediated relations between vaccine confidence/hesitancy and willingness; Pauly et al. identified COVID-19 vaccine hesitant groups from adolescence to late adulthood and explored their motivations for and against vaccination in a nationwide Luxembourgish population, being the vaccination hesitancy higher in the younger age groups; Zilver et al. studied barriers and facilitators for Netherlander pregnant women's choice and motivation regarding vaccination against COVID-19 during pregnancy, verifying that they needed clear, unambiguous information concerning health consequences, particularly for their offspring; and Wang et al. conducted a qualitative survey using Vaccine Hesitancy Determinants Matrix and 5C model to understand and improve Herpes zoster vaccination rates among middle-aged and older adults in China.

### 2.2 Three papers address parents' vaccination hesitancy

Low et al. investigated parents' vaccine hesitancy rates in Malaysia and Singapore, and explored whether these rates were associated with parents' health beliefs, having found that the prevalence of perceived parental vaccine hesitancy was higher in Malaysia; Handayani et al. conducted a study in Indonesia aiming to develop guidance for in-depth interviews for a future qualitative study based on a cross-sectional quantitative study of parents with school-age children and found a significant association between parents' intention to vaccinate their children and the perceived benefits and perceived barriers to vaccination; and Suragh et al. conducted a study on vaccine hesitancy among USA white parents with higher education and socioeconomic statuses, showing they had reliance on information from specialized doctors and scientists, distrust in public health authorities, high risk perception of COVID-19 vaccines, and low risk-perception of COVID-19 disease.

## 3 Vaccination among students

Vaccination among students is a critical public health concern, as this population often lives, studies, and socializes in close contact, increasing the risk of infectious disease transmission (6). Students may face unique barriers to vaccination, including lack of awareness, limited access to healthcare services, or misconceptions about vaccine safety and necessity (7). Understanding the specific drivers and challenges of vaccination in student populations is essential for developing effective strategies to improve uptake and prevent outbreaks in educational settings (6). The seven papers in the *Vaccination Among Students* section are distributed across two Research Topic:

### 3.1 Four papers address students' perceptions about vaccination

Simonovic et al. compared USA and Israeli college students' vaccine attitudes, emotions, and behavior, having found that Israeli (vs. American) participants reported higher perceived ambiguity, worry, fear, and anger, and lower perceived severity; Song et al. explored the circumstances of college students in China's ethnic minority regions concerning their awareness, attitudes, and practices related to the HPV vaccine, aiming to provide a scientific basis for future health education and HPV vaccine promotion; Li et al. investigated the awareness of HPV and its vaccine among college students in Zhengzhou (China), and to explored the factors influencing their awareness of HPV vaccine, to understand college students' willingness to receive the vaccine; and Schlopsna and Scheersoi conducted a study involving interviews with Germany secondary school students, experienced educators, and vaccination experts, showing focal areas of students' interest in the topic and the value of involving students in lesson planning.

### 3.2 Three papers address school teaching on vaccination

Gaspi et al. identified and analyzed Brazilian primary school children's social representations of vaccination, showing the need to prevent children's fear of needles at the first vaccination experience and the importance of discussing the subject of vaccination in science teaching; Kwella et al. conducted a study in German school biology classes to enable students to engage in an in-depth examination of the vaccination complex socio-scientific issue and promoted enhancement of their argumentation and decision-making skills; and Martínez-Pena et al. presented a design on vaccination for secondary education teaching, in Spain, to develop students' integral understanding of vaccines role and to apply critical ignorance as part of criticality to avoid vaccine hesitancy and raise trust in science.

The research presented in these 29 papers published in the Public Health Research Topic “*Vaccine education and promotion”* came from 15 countries of the various continents, demonstrating how live and pertinent is this field of research worldwide, presenting different approaches and perspectives that enrich this large, contemporary and relevant research field, which is still open to wide-ranging and diverse future research.
